# Job demands and resources perceived by hybrid working employees in German public administration: a qualitative study

**DOI:** 10.1186/s12995-024-00426-5

**Published:** 2024-07-19

**Authors:** Leonie Jaß, André Klußmann, Volker Harth, Stefanie Mache

**Affiliations:** 1 Institute for Occupational and Maritime Medicine (ZfAM), University Medical Center Hamburg-Eppendorf (UKE), Seewartenstraße 10, Hamburg, 20459 Germany; 2 Department Health Sciences, Competence Centre Health (CCG), Hamburg University of Applied Sciences (HAW), Ulmenliet 20, Hamburg, 21033 Germany

**Keywords:** Hybrid work, Public administration, Job demands, Job resources, Support needs, Interviews, Health promotion

## Abstract

**Background:**

Hybrid working arrangements that combine remote and office work are on the rise. Although hybrid work has been associated with mental health benefits in employees, challenges in the transformation to hybrid persist particularly in public administration organizations which have been connected to a pronounced culture of presence and inadequate technical infrastructure. Further evidence on the link between hybrid working conditions and employee health is needed. To support the establishment of healthy hybrid working conditions, this study aims to identify employees' job demands, resources and support needs in public administration.

**Methods:**

Semi-structured interviews were conducted with *N* = 13 employees who work hybrid in public administration organizations in Northern Germany between February and May 2023. Interviewees were asked about their perceived job demands, resources, and support needs in hybrid work. The data was analyzed in a deductive-inductive approach of qualitative content analysis, primarily supported by the job demands-resources model as a theoretical framework.

**Results:**

Several job demands, e.g., an increase in work and meetings, and resources such as personal freedom and responsibility, were identified in the context of hybrid work. A multitude of the reported job resources and demands relate to work organization and social relationships. The results disclose discrepancies between participants' experiences of job demands and resources, underlining the subjectivity of employees' perceptions of hybrid working conditions. Interviewees' support needs for hybrid work also varied, encompassing structural-level aspects such as increased acceptance and promotion of hybrid work in the organization as well as behavioral-level aspects, for instance, strategies and self-discipline for boundaries and structure.

**Conclusions:**

This study provides a first comprehensive overview of the job demands, resources and support needs in hybrid work in public administration. This study builds an important basis for further research to understand the impact of hybrid working conditions on health-related employee outcomes. The identified support needs provide a valuable point of reference for health-promoting hybrid working conditions which public administration employers should begin establishing as early as possible in the ongoing transition to hybrid work.

**Supplementary Information:**

The online version contains supplementary material available at 10.1186/s12995-024-00426-5.

## Background

Companies and employers are experiencing a trend towards hybrid working arrangements. Data from a German survey reports that in early 2023, over 60 percent of small, medium-sized, and large businesses in the private and public sector enabled almost their entire workforce to work on a remote or hybrid basis [1]. In the aftermath of the COVID-19 pandemic, hybrid work has increasingly been described as the "new normal" (e.g., [2–5]). While it appears that the combination of remote and office work has become a standard form of work, the transformation process to hybrid work in public administration organizations within Germany was shown to be accompanied by particular challenges [6–8], making it important to investigate in more detail the hybrid working conditions in such work contexts.

Since before the beginning of the global COVID-19 pandemic in late 2019 and early 2020, there has been a rise in flexible and alternative working arrangements which are characterized by flexibility in the employment relationship, the scheduling of work, and the work location [9]. In the course of the COVID-19 pandemic, an abrupt trend towards remote work occurred, which prompted even those employees to work from home, who worked in professions where flexible working arrangements were previously uncommon [10]. Working from home was shown to offer advantages such as work-life balance, improved work efficiency, and greater work control [11]. However, there are also disadvantages connected to working from home, such as work and career uncertainties, social and professional isolation, and inadequate work equipment [11, 12]. Further evidence points to a decline in employees' mental health due to isolation and loneliness connected to working from home [13]. The concept of hybrid working arrangements, on the other hand, has been portrayed as promising to foster socialization and informal communication between employees. Loneliness and isolation could be reduced, and employee mental health thus improved [13]. Hybrid work seemingly represents an ideal compromise by avoiding isolation in remote work while still maintaining flexibility. In hybrid work, employees could benefit from a "best of both worlds" by experiencing the combined advantages of both the remote and the office working arrangement [14].

### Definition of hybrid work

To our knowledge, there is no existing condensed definition of hybrid work yet. However, four characteristics can be derived from the literature. Within the scope of this paper, hybrid work is understood as a "fluent spectrum" with remote work and office work on the two ends [15]. According to this understanding, employees are allowed to spend part of their working time remote. The actual proportion of either can be between zero and 100 percent so that there is no obligatory amount of time that has to be spent at a certain site – only the possibility to change sites in general. Therefore, parts of the workforce could work full-time in the office or remote but would still participate in the hybrid way of working [15, 16]. Further aspects that characterize hybrid work are that the work executed remotely does not mandatorily have to be performed from the home office but can, depending on the employer, be performed from anywhere else than on-site [17]. Team members in hybrid work settings are at times or always distributed among different locations [18] and are dependent on virtual as well as face-to-face collaboration [19].

### Implementation of hybrid work

In work trend publications, the proportion of hybrid workers ranges from 38 percent globally (across 31 countries) [20] to 18 percent across the European Union [21]. In Germany, 74 percent of employed people prefer hybrid working arrangements over exclusively working either from home or on-site [22]. Survey data from mid-2021 and early 2022 shows that the average number of days per week that employees spend working from home is at 1.4 within Germany [23]. Employees with the opportunity to work hybrid were previously shown to have significantly lower attrition rates and significantly higher values in self-reported satisfaction measures (e.g., job recommendation, work satisfaction, life satisfaction, and work-life balance) as opposed to employees who work fully on-site [24]. Odds for having negative self-rated mental health are higher in employees who work exclusively on-site and from home compared to hybrid workers [25].

In public administration organizations, however, the implementation of hybrid work is faced with special prerequisites. Public administration has been connected to a strong culture of on-site presence as a survey by Neumann et al. [8] from Germany shows. Around half of the surveyed employees from public administration institutions for instance agreed that not being on-site would be a hindrance to the career or that presence is important to show that work is being done [8]. A pronounced culture of presence, in the sense that supervisors attach great importance to the presence of their employees, can be a decisive factor among employees to not work remotely [6]. A Germany-wide survey from 2016 showed that public administration employees' work activities could in 60 percent of cases in principle be carried out from home [26]. In practice, pre-pandemic data, however, shows (partial) home office work in public administration was implemented in eight to 16 percent of cases [7, 26]. Along with the pandemic-related restrictions in early 2020, this number increased to 74 percent. This transition in public administration was reported to be accompanied by challenges such as transforming the technical infrastructure, digitizing work processes, and insufficiencies in technical equipment [7].

The increasing spread of hybrid work in public administration therefore represents a process of profound organizational change, posing challenges for employers and employees. The mental health of employees has previously been positively associated with hybrid working arrangements [24, 25] and deserves particular consideration for a successful transformation process towards hybrid work. Designing health-promoting working conditions [27] in support of mental health in hybrid work necessitates a prior analysis of current working conditions and consideration of work characteristics that impact employees' health in both negative and positive ways.

### Theoretical framework

The job demands-resources (JD-R) model, introduced by Demerouti et al. in 2001, is a theoretical occupational stress model that can be applied in different work contexts and that postulates how specific work characteristics can positively or negatively affect employees. Different physical, psychological, social, or organizational factors can be classified into two corresponding categories: job demands and job resources [28–30]. Job demands entail persistent physical and/or psychological efforts and can strain employees [29], e.g., a high work pressure [28]. Job resources can help employees reduce demands, support the achievement of work goals, and stimulate individual growth and development [29]. Job resources can, e.g., consist of autonomy or growth opportunities [28]. Depending on the context and their reciprocal balance, job demands and resources can lead to either a health impairment process or a motivational process and, in the course of this, predict different motivational (e.g., work engagement, job performance, or organizational commitment) and health-related outcomes (e.g., burnout, absence duration, or well-being) [28]. As part of this, the model also considers the interaction between job demands and resources which can affect the consequences for the employee. Specific job resources can, for instance, buffer the effect of job demands [28]. Qualitative research based on the JD-R model enables an initial identification of the job demands and resources and support needs of employees that are prevalent in specific work contexts [31] such as hybrid work. The model can thus provide a foundation for potential risks at the workplace to be eliminated and enable the establishment of health-promoting hybrid working conditions to ensure the health and well-being of employees [27]. The JD-R model has also been used to inform workplace risk assessment approaches by various occupational health and safety stakeholders [28].

In Germany, mental stress of employees is mandatory to include in workplace risk assessments under the Occupational Safety and Health Act (Sect. 5 (3)). The Joint German Occupational Safety and Health Strategy (GDA) as a central alliance for occupational safety and health in Germany [32, 33] published and regularly renews recommendations for assessing the risk of mental stress at the workplace [34]. Herein, six domains of working conditions are to be considered and modified, if necessary, to avoid risks due to mental stress: 1. *work contents and tasks*, 2. *work organization*, 3. *work time*, 4. *social relationships*, 5. *work equipment*, and 6. *work environment* [34]. These domains will refine the study's analysis and classification of the job demands and resources identified in hybrid work.

Based on job demands and resources specific to a respective work context such as hybrid work, appropriate measures can be derived to improve working conditions and realize workplace health promotion [30]. Respective measures can be introduced at the behavioral and structural level. Structural-level strategies relate to a change in working conditions in the company or individual company divisions. They necessitate a transformation of the organizational or leadership culture and focus on the respective working conditions [35, 36]. Behavioral-level strategies relate to a change or avoidance of harmful work behaviors in individual employees [35, 36]. Support needs and opportunities for improvement on both the behavioral and the structural level form a second focus of this study's analysis.

### Current state of research: working conditions in hybrid work

Findings of the current literature that address working conditions in hybrid work reveal benefits as well as downsides of hybrid work. Regarding *work contents and tasks* in hybrid work, challenges may arise due to insufficient qualifications of supervisors for navigating their tasks and responsibilities in hybrid work [18]. Further findings also address a decrease in employees' autonomy [37] whereas others, on the contrary, also report on the perception of more control and autonomy [38]. Several studies address aspects of hybrid *work organization* [18, 37, 39–41]. Potential job resources such as such as flexibility, opportunities for socializing and collegial support and increased productivity were reported [18, 37, 39, 41]. The latter has, along with employee motivation and engagement, however, also been mentioned to be lacking in hybrid work [37, 40]. There are indications that hybrid work negatively affects *work time* [37, 40, 42, 43], e.g., by increasing working hours and weakening boundaries between work and private life. Nevertheless, advantages were also pointed out [18, 37, 39, 41], such as an improved work-life balance. In addition, workplace-based *social relationships* were suggested to be linked to different potential job demands in hybrid work [18, 37, 38, 44, 45], in cases revolving around social isolation or inequalities between employees depending on whether they work from home or on-site. Hybrid work, however, also offers the potential to strengthen relationships by fostering employees' feelings of belonging or facilitating supervisors' commitment to their employees [37, 41]. Other potential job resources relate to the hybrid *work environment*, such as fewer work interruptions [39] and the availability of different workplaces to match to respective work tasks [37]. Regarding *work equipment*, difficulties in hybrid work, as apparent in the literature, relate to a lack of ergonomic features or inadequate equipment for meetings in a hybrid format [18, 37]. With digital work being an immanent part of the hybrid workplace, further associated job demands might lie in e.g., a constant availability or inconveniences in information and communication technology [46].

Hybrid working conditions have already been investigated in corresponding literature, however, largely with a focus on specific aspects. There therefore remains a need to comprehensively research both the job demands and the resources while at the same time considering the broad variety of areas or domains in hybrid work that they potentially apply to. Due to the recency of the rise in hybrid work, first-hand experiences of the affected group of employees need to be captured to gain initial insight [47]. Particular relevance goes to applying qualitative research to generate in-depth knowledge on the associated job demands and resources. Although first evidence on hybrid work conditions specific to public administration exists [37], it is important to expand this knowledge in an international context and therefore to obtain evidence from other countries such as Germany. Further relevance goes to obtaining information on future support needs and improvement options for hybrid work directly from the affected target group instead of deriving practical implications solely based on reports on current working conditions.

## Study aim and research questions

The *aim* of the study was to gain exploratory insights into the job demands and resources perceived by hybrid working employees in public administration in Northern Germany and identify corresponding support needs. To address this aim, the two following *research questions* were proposed and to be answered by conducting and analyzing qualitative interviews with hybrid working employees in public administration:

1. What job demands and resources do employees in public administration perceive in the context of hybrid work?
2. What support needs and opportunities for improvement for hybrid work do employees in public administration perceive on a behavioral and structural level to ensure health-promoting working conditions?

## Methods

### Study design

An exploratory qualitative research approach was chosen to gain initial insight into the research subject. Exploratory studies are particularly suitable for investigating research gaps and offer openness and flexibility towards the subject of interest [47].

The target group of this study was inquired based on the method of the problem-centered interview (PCI) [48]. The collected interview data was then analyzed following Mayring’s [49] approach of qualitative content analysis. The reporting of this study follows the Consolidated criteria for reporting qualitative research (COREQ) [50]. The study was approved by the Psychologic Ethics Committee of the University Medical Center Hamburg-Eppendorf, Germany (reference number: LPEK-0167).

### Sample selection

The study population of interest was recruited via snowball sampling. Inclusion criteria for participants in this study involved 1. employment or civil servant position in public administration, 2. being aged at least 18 years, and 3. working hybrid according to the defining characteristics provided in the background. Via snowball sampling, participants were recruited through already existing contacts to people from the study's target group who referred the researcher to further contacts from the target group. Referral can be repeated in several stages until enough participants or the required sample size has been reached [51]. The recruitment was carried out via telephone and e-mail and the snowballing procedure contained one to three stages, varying per participant. The employees were recruited from five different public administration bodies in a big city of Northern Germany, and, within these, also from different departments. To achieve an adequate sample size for the study the sampling procedure followed the principle of data saturation. Participants were recruited and interviewed to the point where no new themes and contents arose anymore, indicating an optimal time point for analysis of the interviews [52, 53]. Data saturation was achieved at a sample size of *N* = 13.

### Data collection

All previously recruited persons voluntarily agreed to participate in the interviews. The field time of the interviews was from 02/13/2023 to 05/12/2023. The target group was inquired following the method of PCIs [48] by the first author of the study. The interviewees participated either from their offices on-site, from their home office, or from home within their free time. The interviews were conducted in German and took between 30 and 45 min. Audio recordings of the interviews were taken under the consent of the participants for posterior transcription of the content.

PCIs are useful for capturing subjective perspectives and experiences of participants about a specific problem in an unbiased way in order to gain a better understanding and develop solutions. More precisely this is achieved by a combination and flexible use of communication strategies which generate storytelling and strategies aimed at generating comprehension [48]. An interview guideline supported the PCIs. The structure and topics can be found in more detail in the additional files (see Additional file 1). The guide provided for an introductory phase before the interviews in which the participants were briefly informed about the study, the interviewer's reason for conducting the study, and received a theoretical introduction to the topic of hybrid work. Afterward, they were asked for basic data on demographics, work experience, and job-related information using a standardized short questionnaire to obtain an overview of sample characteristics. The introductory phase was followed by two main phases of the interview on 1. job demands and resources in hybrid work and 2. support needs and opportunities for improvement for a design of health-promoting working conditions in hybrid work.

### Data analysis

The totaled ten hours of audio recordings of the interviews with *N* = 13 participants were transcribed verbatim. The collected data was then analyzed following Mayring’s [49] approach of qualitative content analysis by L.J. In the case of this study, deductive category formation (content structuring analysis) was combined with inductive category formation [54]. While the deductive approach involved a theory-based derivation of categories, inductive categorization was used to include categories that were not based on existing theory. The analysis was performed using MAXQDA 12 [55].

The JD-R model [28–30] served as the theoretical framework to dichotomize the job demands and resources that employees in public administration perceive in the context of hybrid work. The categories subordinate to each the demands and resources were formed deductively based on the six domains of the GDA: 1. *work contents and tasks*, 2. *work organization*, 3. *work time*, 4. *social relationships*, 5. *work equipment*, and 6. *work environment* [34]. Organizational resources were included as an additional inductive domain subordinate to the job resources. In the context of this study, they were understood as additional services or interventions provided by the employer that are related to the workplace but do not directly relate to the individual's work. The specific factors mentioned as job demands and resources in the interviews were then formed inductively, derived from the statements by the interviewees to ensure appropriateness to the individual working experiences of the participants. For an elaboration of the support needs and opportunities for improvement in hybrid work as perceived by the participants, a deductive classification of categories into structural- and behavioral-level [35, 36] needs was carried out. To illustrate the results as presented in the following section, select quotes from the interviews were translated from German to English.

## Results

### Sample description

The characteristics of the participating employees are shown in Table 1. The employees were from five different public administration bodies in a big city of Northern Germany, and, within these, also from different departments. Almost half of the study population (*n* = 6) reported having their focus of work in the field of information technology or digitalization. Other work focuses that were represented in the sample included, e.g., workplace health promotion, business, or public health service. Apart from one person none of the participants declared working in a leadership position. Four other persons stated that they had partial leadership responsibility either at a lateral level or in the case of substitution.

**Table 1:** Participant characteristics of hybrid working employees in public administration (*N* = 13)

Variable	n	%
*Gender*		
Male	5	38.46
Female	8	61.54
*Age*		
21–30 years	2	15.38
31–40 years	4	30.77
41–50 years	6	46.15
51–60 years	1	7.69
	*Mean: 40.54 years; range: 28.00 to 55.00 years*	
*Working experience in current position*		
< 1 year	1	7.69
≥ 1–3 years	9	69.23
> 3 years	3	23.08
	*Mean: 3.79 years; range: 0.75 to 21.00 years*	
*Weekly working hours*		
< 20 h	1	7.69
≥ 20–35 h	2	15.38
> 35 h	10	76.92
	*Mean: 36.50 h; range: 19.50 to 40.00 h*	
*Estimated percentage of work time dependent on communication with others (vs. time working alone)*		
< 50 percent	2	15.38
≥ 50–75 percent	11	84.23
> 75 percent	0	0.00
	*Mean: 53.08 percent; range: 30.00 to 70.00 percent*	
*Estimated percentage of work time spent in the office (vs. time spent remote)*		
< 25 percent	3	23.08
≥ 25–50 percent	9	69.23
> 50 percent	1	7.69
	*Mean: 33.72 percent; range: 10.00 to 55.00 percent*	

### Job demands in hybrid work

In accordance with the JD-R model [28–30], different job demands and resources in hybrid work were perceived and reported by the participating employees. An overview of the different factors individually elaborated upon in detail in the subsequent sections is provided in Table 2. The column on the left indicates how the respective job demands and resources were assigned to the six domains of the GDA [34] and, as a seventh domain, to organizational resources which we were able to identify as an additional type of job resource in hybrid work.

**Table 2:** Overview of job demands and resources perceived by hybrid working employees in public administration

	**Job demands in hybrid work**	**Job resources in hybrid work**
Work contents and tasks	▪ Increase in work and meetings ▪ Difficulties in training new employees ▪ Monotony	▪ Personal freedom and responsibility ▪ Increase in knowledge and competence
Work organization	▪ Rejection of hybrid work ▪ Dual responsibility in private and work life ▪ Limited talking culture ▪ Limited communication in digital and hybrid meetings	▪ Adaptability ▪ Simplicity in coming together ▪ Successful communication and collaboration ▪ Joint office attendance times ▪ Sense of efficiency and productivity
Work time	▪ Limited flexibility ▪ Lack of boundaries and structure	▪ Saving travel distances to the office ▪ Preservation of boundaries and structure ▪ Compatibility with private life
Social relationships	▪ Difficulties in finding connection with others ▪ Lack of social contact and exchange ▪ Managers losing touch with employees ▪ Pressure in office attendance	▪ Maintenance of personal contact ▪ Healthy distance in relationships ▪ Maintenance of interpersonal relationships ▪ Trust and support in the team ▪ Trust and support from managers
Work equipment	▪ Misuse of communication- and cooperation software ▪ Missing or inadequate technical and ergonomic equipment	▪ Availability of technical and ergonomic equipment ▪ Purposeful use of technology and software
Work environment	▪ Occupancy of offices during online meetings	▪ Working free of distractions ▪ Interplay of office and remote work atmosphere
Organizational resources	–	▪ Workplace health management and health promotion offerings

#### Work contents and tasks in hybrid work

##### Increase in work and meetings

In relation to work contents and tasks in hybrid work one perceived job demand consisted of the increase in work and meetings. Participants described that work in itself and communication routes had become significantly faster. Due to hybrid and digital possibilities employees experienced a lower threshold for the arrangement and a considerable increase in the number of (digital) meetings. There was also a tendency for meetings to occur more spontaneously, disrupting ongoing tasks as colleagues or managers would initiate impromptu calls or schedule meetings without prior notice:

*“[…] there have also been moments when you are of course absorbed in your work and then you get a call 'can you say something quickly about this?'. I think this transition has to happen very quickly […] and can of course also have an overburdening effect.” (participant 7, male, age 31–40)*

The digitization of meetings eliminated physically moving from one conference room to another in between meetings, resulting in insufficient opportunities to come to rest. To certain interviewees, the content of online meetings appeared to be very compressed. Because in digital meetings, informal exchange was lacking, they also required higher levels of concentration. Due to the shorter communication routes, employees also felt confronted with higher time pressure in providing responses and work results. According to one participant, the expectations for processing times, primarily held by higher management levels, were too high:

*"[…] I think it's also simply a matter of digitization, so it's expected that you answer quickly. But that, that starts with the big and small queries that you have to answer within a certain period of time […] so the pressure from the top is simply too big in my opinion." (participant 12, female, age 41–50)*

##### Difficulties in training new employees

Participants also reported on second-hand experiences of colleagues who had started joining the authority during the transition to remote or hybrid work and who could therefore experience difficulties in getting accustomed to the day-to-day work. Especially if they were not yet familiar with procedures specific to work in public administration, the frequent absence of colleagues able to provide on-site support could restrict the familiarization with workflows:

*“[…] I feel a little sorry for the colleagues who have started working with us in the last few months because it is of course much more difficult for them to get on board.” (participant 2, female, age 41–50)*

##### Monotony

One participant described a kind of monotony that took hold, especially on days spent in the home office as part of hybrid work. Since, among other things, the unplanned conversations that otherwise take place in the office were eliminated in the home office, a lack of creativity and intellectual stimulation emerged:

*“[…] so, this large-scale, constantly meeting online is just, it's just not that much fun. [...] I always come back from the office much more exhilarated than I am after a day spent at the home office. [...] [S]omehow, I'm in a better mood.” (participant 2, female, age 41–50)*

#### Work organization in hybrid work

##### Rejection of hybrid work

Another demand that employees experienced is the lack of acceptance and endorsement of hybrid work from the management levels. From the perspective of some of the interviewees, managers often seemed to exhibit a more critical view toward hybrid work, especially the component that concerns working from home and, e.g., frequently insisted on meetings being held on-site. People at the management level were described to tend to decide for themselves how far they enabled hybrid work, despite a rule from the public employer that, according to participants, allowed 60 percent of the working time to be spent working from home and 40 percent on-site (60–40 rule):

*“That [hybrid work] is actually not supported throughout the whole organization. I think that's a great pity […] that every boss is his own small king, so to speak, and can decide for himself whether he thinks it's good or not.” (participant 12, female, age 41–50)*

##### Dual responsibility in private and work life

One participant also described that with the ability to work hybrid, she experienced greater conflict between her private life responsibilities and work – feeling more torn between the demands of both. Her knowing that it was in theory possible to work in both places the home office and on-site, hybrid work reinforced emotional dilemmas such as when her child was sick, making the decision to travel to the office more difficult:

*“And I also understand the curse aspect, of course, so you're just a bit more torn when others place demands on you at home, but because my daughter is in daycare, yes, I usually only have these demands when she's sick.” (participant 9, female, age 31–40)*

##### Limited talking culture

According to the participants, the distribution of work locations within the team comes with difficulties in coordinating mutual professional exchange and limited the talking culture. Participants experienced this to be particularly prevalent when pandemic-related home office restrictions were introduced at their workplace. Difficulties in coordination led to insecurities towards their own work results, due to the lack of communicative confirmation from others. Although digital communication options existed, co-workers were not always available. Some described that not everyone within their teams used the availability status functions offered by video conferencing software increasing the barrier for spontaneous calls. A lack of coincidental encounters and opportunities for networking with co-workers and other employees eliminated possibilities for small talk and creative conversations:

*“[…] networking, which is also an essential part of working nowadays, is also difficult because, […] meeting in the hallway, with coffee in the kitchenette, this little small talk that gets lost, and I think that's just something very, very important, which actually also defines the working atmosphere or integrity.” (participant 7, male, age 31–40)*

##### Limited communication in digital and hybrid meetings

Digital meetings remain an immanent part of hybrid work, e.g., as there is less overlap in office attendance between co-workers. Participants experienced demands in relation to associated communication difficulties. Employees described having a mental barrier towards addressing their concerns, especially in larger group meetings, as they felt under observation and unable to interpret other participants' reactions (e.g., approval vs. disapproval). Spontaneous reactions would also only show up to a limited extent:

*"And what I also find difficult is that in these online meetings, and that's why I think they are [...] not suitable for everything because they don't allow spontaneous reactions. So even if everyone has their camera on, it's like, when you're sitting in a group, sometimes people simply make a noise, or someone says something [...] and then everyone starts laughing. You don't hear that at all [in online meetings] because everyone is so disciplined about turning off their microphones so that the meeting isn't disrupted." (participant 13, female, age 41–50)*

One participant experienced that when people from different departments or professions came together in online meetings, they tended to insist on their own opinions more strongly. This would result in difficulties in developing common ground, compromises, and mutual trust.

In hybrid meetings, part of the participants meet on-site and another part joins online. In this context, one participant criticized how hybrid meetings often followed old approaches of conversing in meetings on-site instead of finding new solutions. When joining a hybrid meeting online, participants felt excluded from the people participating on-site and were unable to sense the mood within the conference room. Interviewees deemed it was much easier for the people who are joined on-site to interact with each other while online participants' contributions were rather disregarded in such meetings. In addition, sometimes the people on-site shared the same technical equipment so that only one person in the center of the camera was visible. Due to the mentioned disruptions, one participant preferred meetings to either take place exclusively online or exclusively in presence:

*“And overall, I'm not a big fan of hybrid events because I always feel that when people are sitting in the room, those who are predominantly dialed in always come up a tad short, so I always prefer either completely online or completely on-site rather than hybrid events.” (participant 3, female, age 31–40)*

#### Work time in hybrid work

##### Limited flexibility

Regarding the allocation of work time in hybrid work, participants of the interview study reported restrictions in flexibility. In line with the demanding rejection of hybrid work, part of the people in management positions would frequently insist on holding meetings on-site, in cases violating the 60–40 rule, therefore restricting one participant to freely organize his work time he spends either at home or the office. Another participant also mentioned that core time regulations (a specific period in the middle of the day at which everyone has to work) also limit the flexibilization and organization of work time:

*“[…] if you are really supposed to work freely when you want or if you really want to take advantage of these benefits, […] if you already work from home, it would be good to be a bit more flexible overall. And not to be tied to these six hours [of core time].” (participant 6, male, age 21–30)*

##### Lack of boundaries and structure

Participants found it difficult in hybrid work to separate their private life from work life and structure their days:

*“I think what is also important is that some people, for some people, they simply need a clear path. And of course, they get lost, or it becomes more confusing because one must also organize the day by oneself somehow, or organize it more intensively.” (participant 7, male, age 31–40)*

Because of the lack of structure in the day, some tended to work beyond their normal working hours. For some, this was the case especially when working from home where there is no physical separation from work. For one participant there seemed to be a difference when working on-site as, e.g., the computer was left at the office or there was still time needed for the commute home. One employee admitted that this had also been the case before the introduction of hybrid work, e.g., as many were still reachable on their mobile phones after work – however, with hybrid working these boundaries seemed to have blurred more, and a fixed daily structure has receded further into the background. It was also reported that it was difficult to take lunch breaks, especially in the home office. For one employee, taking a break was sometimes not even possible, as meetings were scheduled during lunchtime. Others would take their lunch break directly at their workstation and in the meantime continue working.

#### Social relationships in hybrid work

##### Difficulties in finding connection with others

Regarding social relationships difficulties arose in finding connection with others. Participants described second-hand experiences of new colleagues. Similar to the difficulties with training, participants reported that new employees had difficulties socializing and growing into the team as co-workers do not see each other on-site regularly:

*“[...] I feel a little sorry for the colleagues who have started working for us in the last few months because it is of course much more difficult for them to even get started. [...] Or even to grow into the team or the department, that is, I think, very difficult. And I would also say that I still haven't gotten to know my colleagues who aren't on-site very much […].” (participant 2, female, age 41–50)*

In online conversations, informal exchanges between colleagues to connect tended to fall away because of a lack of time. Before hybrid work, on the other hand, people went to the office every day and therefore had a chance to become acquainted with almost everyone on-site.

##### Lack of social contact and exchange

The lack of social contact and exchange applied to informal exchanges between colleagues, which affected some participants' ability to create a counterbalance to the focused and primarily digital work in hybrid arrangements. Small talk that used to take place, e.g., over lunch or coffee, has become much less frequent, especially since there is less overlap in office attendance in hybrid work. Spontaneous calls to colleagues aimed at informal exchange also come with the barrier of uncertainty as to whether the other person has the corresponding time capacities. According to one participant, the social circle and support that once existed within the work context would disperse:

*“[…] the workplace represents a large social circle, of course. And this hybrid work has also diluted that to a certain extent. I think there are some people who are good at managing this, and other people, especially our older generations, who have difficulties in shaping or participating in this change, and for whom this social circle is obviously crumbling.” (participant 7, male, age 31–40)*

##### Managers losing touch with employees

One participant criticized that due to infrequent exchanges in hybrid working, managers would no longer be able to track their employees' well-being, which possibly would be different if they were to talk in person on the regular. Managers would not be able to inquire about employees' feelings:

*“[...] you have to know your own employees. If you only hear or talk to them once a week, perhaps via Skype cam, then you simply lose a bit of something. If someone is in a bad mood [...], on a day in the office, you are more likely to notice it than if you talk to someone briefly via Skype.” (participant 7, male, age 31–40)*

##### Pressure in office attendance

Employees referenced a pressure to attend the office which came from their managers as well as co-workers who still deemed it important to work on-site, partially out of habit. When the office attendance advocates worked on-site themselves and planned meetings with a requirement to join physically, the pressure to show presence also increased for other employees. Some participants thus felt restricted or deprived of the remote working opportunities to which they are entitled in hybrid work. Furthermore, one employee considered the people who are on-site more frequently to receive more advantages and attention than those who work from home:

*"And my perception is that those who are there in person still have a little bonus: 'Hey, you came to the office today'. And that those who don't participate [in person] are left behind a little bit. That this technological barrier is also used as a means of sanction." (participant 5, male, age 41–50)*

#### Work equipment in hybrid work

##### Communication- and cooperation software

A misuse of communication and cooperation software in digital hybrid work, referred, for instance, to the fact that there seemed to be insufficient licenses, limiting the variety of video conferencing software. When meeting with external parties on certain video conferencing platforms, employees were often dependent on receiving their digital invitations. Even for internal meetings, participants mentioned how chaos was caused due to different rights of use between departments of the authorities, e.g., due to data protection reasons. Two other participants also complained that co-workers sometimes did not use the availability status feature in video conferencing software, which also impaired communication. One participant also revealed how there are often no standardized digital processes when collaborating digitally on the same task:

*“Exactly, with external offices, that media, such things still have to come in paper form, are then being scanned in, then the information is entered by another office, then you have to look at it in another program, but then you still communicate via email, perhaps." (participant 10, male, age 31–40)*

##### Missing or inadequate technical and ergonomic equipment

One interviewee commented that the technical equipment for hybrid meetings in particular is unfavorable. When several people who are co-located on-site join the meeting and share the same equipment, they participate in the meeting as a singular unit, diminishing their individuality. He highlighted the lack of suitable technical solutions that allow the co-located as well as virtual participants in hybrid meetings to see and hear each other and express themselves equally.

Several employees mentioned that they were hardly provided with technical equipment to work in the home office, e.g., a monitor. It was also described that both in the home office and on-site, employees did not comprehensively possess identical workstation equipment which could impair cooperation in hybrid work, also on an interdisciplinary level. The lack of ergonomic equipment of workplaces was also criticized, especially for work in the home office. Employees were not entitled to receive adequate office furniture or corresponding financial support. One participant saw the fact that some employees resorted to alternative solutions or other furniture as endangering, describing the following:

*“I also remembered that the equipment at home is still an absolute health risk, in my opinion. [...] I also often sit in the living room, I don't have a proper chair, the table doesn't have the right working height per se.” (participant 13, female, age 41–50)*

#### Work environment in hybrid work

##### Occupancy of offices during online meetings

When several people shared an office on-site, this led to problems of two kinds. It was described that if office-sharing employees participated in the same digital meetings at a time, disruptions such as audio feedback, unpleasant noises, or talking over each other were caused:

*“When we are on-site, the whole thing is a bit more difficult. I share an office with a colleague. [...] when we sit in the same conferences at the same time [...] and then talk, and then the audio feeds back and you hear everything twice and you talk over each other. That is quite exhausting.” (participant 1, female, age 21–30)*

Another employee recounted that when he was in online meetings where confidential or private information was exchanged while others were sharing the room on-site, the required openness or transparency in the conversation was interfered with.

### Job resources in hybrid work

#### Work contents and tasks in hybrid work

##### Personal freedom and responsibility

The participants of the study saw a resource within hybrid work in the freedom regarding work activities as well as the organization of work tasks and the time spent on them. They were able to schedule the times when they worked at home more flexibly in hybrid work, except for the core working hours. They also felt more autonomy in individually structuring their daily routines. Some participants also profited from freely determining the days on which they attended the office based on their own needs. One employee addressed how on days when he experienced poor mental health, the home office gave him the chance to navigate through his feelings while continuing to work whereas in times without hybrid work options he likely would have taken a sick leave. Certain employees also praised when independent decision-making was supported or practiced by their managers. Participants also appreciated that they were able to work autonomously within their team and had a lot of freedom in decision-making in the way they organized their work and work tasks:

*“[…] there are some phases where there's less to do, or where you're not quite focused on your projects, and I actually find it quite healthy to be able to be in the home office in such phases, because it simply gives you more freedom in dealing with your own time. [...] Office jobs always have phases or aspects that are less motivating, but it's not hybrid work that causes that, rather in the hybrid format you are actually able to deal with it better, I think.” (participant 10, male, age 31–40)*

##### Increase in knowledge and competence

Participants rated the opportunities to learn new skills in the hybrid work situation positively, especially in the technological area (e.g., for software such as Excel), but also in presentation, controlling, and moderation techniques. This was enabled partly through trainings approved by managers, but also through the support of other colleagues:

*“[...] so the amount of technical knowledge I have actually acquired here is something I didn't have in all those years before because I didn't see the necessity.” (participant 12, female, age 41–50)*

#### Work organization in hybrid work

##### Adaptability

Participants rated openness to communication and change within teams and managers as important. One participant described how his team regularly reflected together and discussed openly ways to communicate in a hybrid format to find the most suitable methods and then also adapt the existing communication channels. To him, it was important that the managers actively showed support and willingness to adapt:

*“This process of thinking about and talking about when and how to communicate is always a topic. […] That is, that was also the announcement from our managers that this can also be adapted time and again and that they are also open to us communicating our needs […].” (participant 11, male, age 41–50)*

Another participant described how she found solutions for upkeeping creative exchange in hybrid work with her co-workers, by finding adapted hybrid solutions such as simple phone calls or drinking a coffee together per video call.

##### Simplicity in coming together

According to several participants, appointments and meetings can be organized much more easily in hybrid work mode. Since digital and hybrid meetings have gradually become the norm, different people could participate more quickly than in on-site meetings, especially if they came from different departments, states, or countries. Issues could be resolved at shorter notice, resulting in quicker decision-making.

*“I would also describe it as positive that you don't always have to schedule, let's say, a big appointment, but that you can simply call someone, get someone to join you, […] and schedule or organize appointments that can be arranged at short notice. […] [W]ith hybrid work, it is easier to connect with people or to connect with them more quickly, yes.” (participant 7, male, age 31–40)*

##### Successful communication and collaboration

In the interviews, there were many reports that exchange and collaboration with colleagues succeeded both in the office and digitally. Both formats offered different advantages that complement each other in the hybrid format. In the office, it was particularly easy to coordinate and resolve questions quickly, e.g., when asking other colleagues for their opinions. Mutual understanding was also easier, since the people that the participants communicated with, and their reactions were easier to judge in person than in digital communication. It was also mentioned that there were more creativity, commitment, and people got to know each other better in face-to-face conversations. In addition, better results and new ideas could be developed in person:

*„[...] if we really want to work out results [...], then it is so much more efficient if you sit down together for two days. So, I had one working group specifically, [...] and we came together again and again, constantly we met online and for several hours [...]. It was all about developing new things and then we met just once in presence over two days and were able to clear everything up.“ (participant 2, female, age 41–50)*

In digital communication, the employees named advantages, such as being able to work through meeting agendas more efficiently. The exchange of information and discussions was facilitated since tools such as screen sharing could be used instead of having to gather around a single computer. One participant felt an increase in professionalism in meetings enabled by modern (collaboration) media. Another advantage was the improved reachability of co-workers, facilitated by messenger and video conference software.

Another positively perceived factor that related to both digital and face-to-face communication in hybrid work was a constant availability of the supervisor. In addition, participants referred to successful contact with co-workers, irrespective of the place of work. It was described by one participant how hybrid work enabled colleagues to inform each other, e.g., about missed calls or letters in the office when others are not on-site themselves. It was also perceived as positive when colleagues informed each other via e-mail as soon as they arrived at the workplace (either on-site or in the home office) so that everyone was aware of each other's presence. Contrary to the perceived demands of hybrid meetings, one participant also felt that hybrid events work successfully.

##### Joint office attendance times

Several participants approved of joint office attendance hours days when all members of the team were to be on-site in order to maintain a face-to-face exchange. In this way, the employees were ensured periods in which they could meet their colleagues in person:

*“Some of the department members have arranged to meet [...] on Thursdays. It's really nice to talk to each other, to go out for a meal together, so that contact doesn't get lost if you are not directly involved with each other.” (participant 6, male, age 21–30)*

##### Sense of efficiency and productivity

In a sense of personal competences, some participants perceived that as digital work had increased due to hybrid work, their efficiency had also increased:

*“[…] my perception is, and I notice this for myself, that through this hybrid work, digital work has increased, and I have actually become more efficient.” (participant 5, male, age 41–50)*

Part of them experienced this increase especially on those days when they worked in the home office as it allowed for more peace and prevented distractions from colleagues. Other participants, on the other hand, felt more productivity and balance when they worked on-site at the office.

#### Work time in hybrid work

##### Saving travel distances to the office

Participants experienced many benefits by eliminating the commute on the days they worked in the home office, thus saving time. They were able to begin working directly in the morning and leave work immediately in the evening. This also allowed for more time for private life activities. In other occurrences, the saved time was also reallocated to work time:

*“[...] what's obvious is that you save the commuting, which means that you practically have more capacities available. Be it that you can sleep a little longer or have a little more time even for work, because you save the commute, so that I see more advantages in that at the moment.” (participant 3, female, age 31–40)*

##### Preservation of boundaries and structure

The interviewed employees described favorable circumstances of hybrid work that provided for more demarcation and structure in the workday. This included the 60–40 rule for a regular obligation to travel to the office, which thus secured routines and continuity:

*“So compared to the times of the pandemic, I would say that hybrid work is really much more pleasant, [...] this obligation to attend the office every now and then, [...] in order to create a bit of continuity in everyday life or a certain routine, so that you don't completely derail, I would say.” (participant 6, male, age 21–30)*

Attending the office several days in a row, so that the work laptop remained in the office, made it easier for participants to switch off after work. Core working hours ensured that employees remained in a reasonably fixed daily structure. Others described that they scheduled meetings to always start a few minutes after the full hour and end before the next full hour to secure breaks in between meetings. In terms of personal competences, some participants were able to allocate their tasks themselves, keep track of their calendar, and cultivate habits:

*“Maybe also a little bit of habit. So, that I have set myself a fixed daily routine. That I said to myself, 'You'll be in the office by 8 at the latest, my dogs will let me know when it's time for midday break, and, yes, the day ends when the last meeting is over,' something like that.” (participant 12, female, age 41–50)*

##### Compatibility with private life

Hybrid work allowed employees to reconcile work with their private lives more. According to participants, this included the individual organization of everyday life, more time for relationships outside of work, and more leisure time in general, e.g., due to fewer commutes. In the home office, household tasks could be taken care of during lunch breaks. Another aspect was easier childcare:

*“[...] so a resource is definitely that I, for example, when my child is sick, can work almost equivalently of course. Otherwise, I would probably have had to call in sick every time or go with him to the [...] pediatrician, so that we would receive a sick note. [...] That is of course a real advantage. I've also been on vacation once and had an important meeting, which I was able to attend while still being on vacation.“ (participant 2, female, age 41–50)*

#### Social relationships in hybrid work

##### Maintenance of personal contact

Compared with working exclusively from the home office, personal face-to-face contact could be maintained in hybrid work. Many participants considered this to be beneficial and a source of satisfaction:

*“[…] this team atmosphere or this, this binding matter between the employees is, I think, very important. And you can only experience or develop this if you really work in presence because then I can simply also perceive small things, be it impressions, emotions, or even smells [...]. And I think that is important [...] for the working world, because otherwise the integrity could be lost, or also the relationship with the profession or with the job.“ (participant 7, male, age 31–40)*

Specific benefits of maintaining face-to-face contact included a more genuine exchange, as employees could address subjects differently than they would over phone or video calls. A better recognition of others' reactions (e.g., laughter) also contributed to this and helped to build trust in the team. Face-to-face contact with colleagues was further essential to compensate for home office days, which, according to one participant could over time have mentally overburdening effects.

##### Healthy distance in relationships

While maintaining regular face-to-face contact was considered important, some participants alluded that increased digital collaboration (accompanied by less face-to-face contact) within hybrid work could also create a healthy distance in work relationships. This was especially the case if, e.g., interviewees did not get along with co-workers or managers on a personal level. Through hybrid work they had more options to detach themselves from these people while maintaining effective collaboration:

*“[...] I'm very collegial and I really enjoy working, I'm an absolute team player, but I don't really have much private contact with colleagues, but that has always been that way. In this respect, [...] for me it is now in this case, it helps, so to speak, the distance to my supervisor, that helps significantly in the cooperation.” (participant 13, female, age 41–50)*

##### Maintenance of interpersonal relationships

It was seen as a resource when within the team, there was an emphasis on getting to know and understanding each other on an interpersonal and not just on a professional level. In distinction to the maintenance of personal contact (i.e. face-to-face contact), this job resource also addresses the cultivation of interpersonal and informal exchanges in digital collaboration. In hybrid work, relationships continued to be cultivated both on-site and online by sharing personal matters. In some cases, extra meetings were arranged for interpersonal exchange. Other reported strategies were spontaneous phone calls. Interpersonal exchange was deemed important so that co-workers would be aware of how others were doing or could provide mutual support. It was seen as positive when managers encouraged and allowed this kind of exchange, e.g., in meetings. For some, hybrid work emphasized the importance of interpersonal relationships, both online and in person:

*“That when we meet in person, we now make a conscious effort to get to know each other personally. So that this personal touch moves a bit more into the foreground and you are not so much focused on the work, rather you have realized: we need each other as people to work together.” (participant 5, male, age 41–50)*

##### Trust and support in the team

Trust and support in the team were contemplated as another important aspect of hybrid work. This included transparency and open communications about the ways collaboration occurs within the team. Participants appreciated working in a team where everyone stood up for each other:

*“[…] this is one of the best teams I've had in all that time. Because we support each other, we put our own needs aside if we have to. This means that vacation time is postponed when someone is sick, or one is available in an emergency, […] there is a great deal of trust that is really important, but also a willingness to stand back from time to time, to both give and take.“ (participant 12, female, age 41–50)*

Reliability in hybrid cooperation was important, also because there was a mutual dependence that everyone fulfills their work tasks, especially when working from the home office or working at different times of the day. The importance of mutual trust, however, seemingly increased in hybrid work, as private and work life became more intertwined (e.g., by attending meetings from home):

*“[...] you also enter the privacy of each person a little bit. Not everyone has the opportunity to have a separate office in the house. And then a child screams or a dog squawks or something, of course, you always have that, and I think that's also a matter of trust. That you are able to give that.“ (participant 12, female, age 41–50)*

##### Trust and support from managers

In many interviews, the trust and support in various ways from the management was deemed important in hybrid work. Since managers also function as a role model, they could convey to employees that hybrid work and working from home are accepted by setting an example. Others also described how their managers demonstrated their support of hybrid work by not exerting pressure in terms of a fixed balance of on-site and home office work (despite attendance rules) so that everyone was able to decide what works best for them.

Participants also deemed it positive when managers paid attention to employees' individual needs by making sure that everyone was provided for in terms of work equipment and by being available (both in the home office and on-site). Participants appreciated when managers encouraged employees to report their needs and were open to finding solutions when problems arose:

*„[…] they are also open to us reporting our needs and I also found that very important, for example, that it was said from the outset: 'If you see a need and you need to talk, then get in touch or let us know and then we'll work it out somehow and see how we deal with it.'.“ (participant 11, male, age 41–50)*

A mutual basis of trust also helped one employee overcome the occasional discrepancies and disagreements in the relationship with her manager and was seen as a source of work satisfaction. Managerial support revolving around professional aspects was rated important as well.

#### Work equipment in hybrid work

##### Availability of technical and ergonomic equipment

Ergonomic equipment, such as a standing desk, as well as technical equipment, e.g., a well-functioning internet connection, were regarded especially important for work time spent in the home office. One participant mentioned how her equipment is very helpful for work, but at the same time recognized how it is a luxury and privilege:

*“I have everything I need. I can move around, I can work standing up, sitting down. I have everything, that is a luxury, I know that. Many people don't have that. Just to buy a desk like that, that's not available everywhere. Therefore, for me personally, everything is great.“ (participant 4, female, age 51–60)*

##### Purposeful use of technology and software

A purposeful use of technology and software enhanced hybrid work and included, e.g., that everyone used the availability status feature in video conferencing software. With the right use of the appropriate tools, digital collaboration seemed in some cases to work more efficiently than on-site, e.g., for giving presentations on screen or working together on a document via screen sharing. Certain digital tools were also used to share knowledge at all times so that everyone was equally informed. Through the targeted use of technology, both when working online and on-site, the professionalism in appointments seemed for one participant to also increase:

*“And for me, the appointments have also become a bit more professional because it's much easier to use modern media in online meetings. So everyone can access [...] one program [...] and work together digitally. That wasn't possible before. And there were flipcharts and so on before, which is good, but I think it's easier nowadays to document the results and hand them out to everyone, or to work on them together later on.” (participant 5, male, age 41–50)*

#### Work environment in hybrid work

##### Working free of distractions

Particularly the home office environment allowed participants more peace and fewer distractions. If there were a lot of calls, participants mentioned they could talk more quietly at home. Especially with tasks that required quietness participants felt they could work more efficiently:

*“[…] when you're in the office – you have that interpersonal communication and all that, it's all totally important – but you're not so isolated. […] [T]here are tasks where I have the feeling that it works just as well without a team, that you can work more concentrated from home.“ (participant 11, male, age 41–50)*

##### Interplay of office and remote work atmosphere

The alternation of office and home office in hybrid work was rated to be beneficial, as the advantages of both locations were preserved. On the one hand, employees experienced quiet at home, but on the other hand, they still maintained personal interactions:

*„If you're just sitting at home, it can be quite a different situation, depending on what you have around you. If you have a family and children, it's certainly quite a different situation, and you're glad to get out, but it's also quite depressing when you're really just sitting there for yourself in your little corner. This mixture, I think, seems to me the healthiest, at least for me. And also the possibility of quickly stepping out onto the large balcony and shortly taking a deep breath for five minutes.“ (participant 6, male, age 21–30)*

#### Organizational resources in hybrid work

##### Workplace health management and health promotion offerings

Many also rated the employer's workplace health management and health promotion offerings positively and helpful in hybrid work. These included, in particular, (digital) exercise programs or yoga breaks:

*“What I think is really cool, which they've had for a while now, is this gymnastics thing there that you can always do, even if you don't do it, but you're always briefly reminded that you should get […] moving." (participant 8, female, age 41–50)*

### Support needs and opportunities for improvement in hybrid work

In the second interview part, the participants reported on the support needs and the opportunities for improvement that they saw in the context of hybrid work (see Table 3). The suggestions aimed at changes on the structural or the behavioral level. Corresponding to the results in the sections Job demands in hybrid work and Job resources in hybrid work, the possibilities on both levels can also relate to an expansion of resources or prevention and reduction of demands.

**Table 3:** Overview of support needs and opportunities for improvement perceived by hybrid working employees in public administration

Structural level	Behavioral level
▪ Acceptance and promotion of hybrid work ▪ Development of mutual understanding and accommodation ▪ More freedom and responsibility ▪ Office attendance regulations ▪ Psychosocial counseling services ▪ Encouragement of greater boundaries ▪ Deceleration of work and meetings ▪ Improvement of workplace equipment ▪ Improvement of technology and media use ▪ Management by objectives ▪ More targeted job advertisements	▪ Creation of balance ▪ Strengthening exchange and networking ▪ Expression of one's personal needs ▪ Increase of joint office attendance hours or days ▪ Training and workshops ▪ Strategies and self-discipline for boundaries and structure

#### Structural level support needs and opportunities in hybrid work

##### Acceptance and promotion of hybrid work

Participants expressed the need for managers to embrace hybrid work in a positive rather than a negative way. Managers should always question whether an obligation for office attendance is urgently needed and reduce the pressure to attend the office. People who work mostly from home should not experience any disadvantages compared to people who are on-site more frequently. Participants would also appreciate the managers advocating that employees have freedom to make decisions in hybrid work. Training or coaching could help to teach managers leadership strategies for the hybrid working environment. It was also noted that as digitality in work will generally increase, hybrid work will remain an indispensable part of the working world which employers necessarily need to move along with:

*“For me, it's actually a cultural change that's taking place, of which hybrid working is actually only one part. And I see it as the employer's duty to develop a strategy [...]. So, whether I like hybrid work or not, in ten years it will simply have to be more present. […] And the work will also become much more complex digitally [...].” (participant 5, male, age 41–50)*

##### Development of mutual understanding and accommodation

The need for the development of mutual understanding and accommodation is primarily related to a change in management style. Managers should monitor more closely their employees' well-being. More open communication and more trust on the part of the managers were desired by participants and could be established by regular consultations between employees and supervisors:

*“And as far as supervisors are concerned, I think we need new models. On the one hand, perhaps [...] really taking a closer look, what do the people look like, how are they doing, and also using things like employee-supervisor consultations to really address this issue. If you're not doing well, you also talk about it.” (participant 13, female, age 41–50)*

Management should further encourage employees to express their needs and support them in taking advantage of psychosocial consulting services. Managers need to be aware of their employees' different needs in hybrid work (e.g., in terms of work atmosphere or communication). If necessary, employees should be encouraged to use new technology and media, e.g., by referring them to appropriate training courses. Mentorships or training for managers on how to best lead employees in hybrid work were also proposed by the participants. Such offers should especially address managers who have been used to work being performed exclusively from the office and for whom hybrid work represents a major change.

##### More freedom and responsibility

For hybrid work, participants expressed a desire for increased autonomy and responsibility in managing their time, selecting their workplace, and handling work tasks. It should be ensured that appointments are not scheduled spontaneously without prior request and that the core working hours or the 60–40 rule could be relaxed. Participants sought for more flexible choices regarding work locations, in the sense of being allowed to work from a variety of locations other than the office and from home. On another note, employees would prefer greater responsibility and trust in task execution with reduced managerial oversight and micromanagement as long as they meet the agreed upon objectives successfully. One participant put it as:

*“[...] as a manager you have to specify the tasks and the goals and see if they are achieved. [...] And I think you must have that confidence that people are not going to tell you all the time 'Oh, I'm so busy' and still have nothing to do. [...] I think exactly that kind of leadership is necessary for hybrid work [...].” (participant 10, male, age 31–40)*

##### Office attendance regulations

On the other hand, the 60–40 rule as a current attendance regulation did not ensure sufficient on-site work in the team for some participants. Managers or team leaders could regulate for greater attendance. E.g., one participant wished to increase the mandatory attendance rate (usually at 40 percent):

*“[…] ideally, I would even reverse the 60-40 rule, meaning that you are really [...] 60 percent on-site, because I believe that the exchange is very important.” (participant 7, male, age 31–40)*

Joint face-to-face time was rated necessary for teams in order to develop commitment, trust, a good team atmosphere, and a bond with each other.

##### Psychosocial counseling services

The participants welcomed the psychosocial counseling services, which, e.g., provide support in the event of professional or private stress, or conflict moderation for teams offered by the employer. Such services were deemed important in hybrid work since the emergence of working from home meant that employees oftentimes had to cope with issues on their own. However, the need was stated for these services to be more easily accessible and low-threshold:

*“I think these problems that arise in the home office, precisely because you don't get out, maybe it would be good if one could find a possibility to overcome these barriers, to somehow get advice from someone with whom you don't work together directly […] I think that would be good if one could somehow make the access easier.” (participant 6, male, age 21–30)*

As one participant stated, many were also not aware of these offers, which caused them to make less use of them. Here, too, managers should show responsibility and demonstrate more willingness to participate. The way such offers were advertised up to this point may have left concerns among other employees, e.g., regarding data protection.

##### Encouragement of greater boundaries

Participants expressed the need for managers to provide more support or encouragement for employees in establishing greater boundaries. More specifically, this should be a leadership goal and the employer should create more awareness around this issue. Managers could encourage the team or department members to schedule fixed breaks in their calendars and not make their calendars publicly accessible. Furthermore, managers could keep track of their employees' working hours and point out the importance of not exceeding those:

*“And that the boss also points it out from time to time, [...] just as a reminder, like 'remember, you only have to work six hours' and [...] 'it's just work and not everything'. There is more to life than work and that they simply have that in mind, that this is a problem and they simply remind you of it.” (participant 11, male, age 41–50)*

##### Deceleration of work and meetings

To counteract the increase in work and meetings (see section Work contents and tasks in hybrid work), the participants suggested incorporating regular breaks between meetings. Furthermore, online meetings should not be overloaded with the agenda and should focus on fewer topics per meeting, especially if the topics necessitate more in-depth discussions. The pressure and expectations from the employer and management levels concerning the speed at which employees process tasks and requests would have to be reduced. One employee stated how she wished more realistic goals to be defined:

*“[…] there has to return a little bit, yes, more reality, so the view for reality: 'how long does it take to answer a small query and to coordinate with all the departments?'.” (participant 12, female, age 41–50)*

##### Improvement of workplace equipment

Many of the respondents would like to see an improvement in workplace equipment. This would involve the provision or subsidization of two fully and equally equipped workstations for employees in the office on-site and in the home office. On the one hand, the employees' needs applied to technology (e.g., monitors or accessories) and, on the other, to office furniture (e.g., office chairs). In addition, a standardization of the (technical) equipment across all workplaces was desired as well:

*“[…] I never know what my counterpart has, and I now realize that I am very advanced in hybrid working and that it sometimes hinders me in my work when I have to deal with people who are of course not yet so advanced because their working equipment is not the same. And I think that would also be nice if that could be improved.” (participant 5, male, age 41–50)*

Other wishes included the provision of technical equipment that is suitable for holding hybrid meetings so that all participants could communicate and see each other without difficulties.

##### Improvement of technology and media use

Participants expressed that the technology and media that were already available should be used more effectively by the authority as their employer. It would be advisable to expand the programs or licenses for collaboration and communication software. Furthermore, digital processes in workflows would have to be improved to simplify work and prevent technical disruptions:

*“[…] the digital processes that we have, they have improved significantly, but they are still not particularly good in some places. There are many media disruptions, places where the interfaces are not very good. […] [T]he degree of digitization of the processes, that you really have simple, well thought-out digital processes, without media disruptions, is not there yet, and that would simply make the work much easier.” (participant 10, male, age 31–40)*

More specific suggestions from participants included the provision of a toolbox for a variety of digital tools that support different activities or the introduction of an option for sending voice messages within a professional context to replace e-mails from time to time.

##### Management by objectives

In some cases, participants expressed the need for a change in management style. Managers should refrain from strictly controlling employees' work processes and put a greater focus on the results achieved by the employees. This could be reached within management by objectives, as one participant mentioned, or other results-oriented forms of managing and evaluating employees' work performance. In this way, the objectives and respective deadlines that are to be achieved could be defined in advance. Employees should then be given the freedom to independently organize their work approach (e.g., concerning choice of location or time management) with managers being trustful in their employees:

*“[…] that you set certain goals or that each person knows roughly what they want to achieve with their work, but that they then also have degrees of freedom [...]. And that you don't have to control the whole time in daily life and don't have to consult at every step, […] and then the work, whether at the office or at home, can be completed well, no matter at what place.” (participant 10, male, age 31–40)*

##### More targeted job advertisements

One participant suggested that job advertisements for positions should be designed in such a way that new applicants know what to expect. In particular, reference should be made to how hybrid work is designed and organized there and what rules exist so that no false expectations arise from new employees when starting the new job:

*“[...] maybe that would also be something to include in the job description, for example, when you advertise a position, that you immediately say: 'home office is possible up to such and such a degree', so that I know [that] from the outset when I am applying for a position.” (participant 12, female, age 41–50)*

#### Behavioral level support needs and opportunities in hybrid work

##### Creation of balance

Several support needs on the behavioral level were voiced by the interviewed employees. One need was a stronger balance between work and private life. This balance should be created by the employees themselves, e.g., by scheduling private appointments in such a way that they are forced to finish work at some point. In addition, more distraction breaks should be taken during work:

*“What I need even more is to take more distraction breaks. So consciously going out during the lunch break. [...] So this digital thing is very beneficial, but [...] there is a shift from analog work to digital. That means I have to make sure that I make more time for analog freedom.” (participant 5, male, age 41–50)*

Also, regular exercise should be ensured at the workplace and employees should make more use of the exercise programs offered by their employer (see section Organizational resources in hybrid work). Joint walks among colleagues could also be helpful in this regard.

##### Strengthening exchange and networking

Participants expressed a desire for more, regular exchange (both digital and face-to-face) between colleagues, to sit down together and talk without obligation. Although respective offers were already provided by the authority, only a few people took advantage of them. One participant stated that his suggestions had not received a lot of positive attention so far:

*“I just think it would be great if we could really establish something digitally and [...] just exchange ideas once a week for ten minutes or so. [...] I've addressed that several times now, that I would like to see that happen, but nothing has happened yet. I think the rest is not that interested in it.“ (participant 1, female, age 21–30)*

The importance of offering low-threshold events was emphasized, as well as that events should not be too obligatory, as this could rather lead to rejection from employees. Some participants, however, also rated company events or teambuilding activities as important and meaningful and would like to see them occur even more frequently.

##### Expression of one's personal needs

In hybrid work, employees should have the confidence to express their own needs, e.g., about the way the team communicates internally or the desired frequency of home office days. This culture should also be practiced together in the team and encouraged by managers:

*„Perhaps it's not so easy for some people to express their needs. I think it's very important to have the courage to do that. [...] And also the fact that it is important and okay to express this must, I think, first be practiced a bit within the team.” (participant 11, male, age 41–50)*

##### Increase of joint office attendance hours or days

In accordance with some participants that appreciated joint office attendance times (see section Work organization in hybrid work) other participants also found it important and saw an improvement need in so far that certain days in the week or month should be agreed upon in the team, on which everyone tries to work on-site so that they can regularly see each other face-to-face:

*“Or also, I think it's also important to set up joint team days, to really show that 'okay, the project team definitely comes together on a Wednesday', for example [...]. [T]hen the probability is obviously greater that people are there, because, for example, yesterday I was also in the office, and it was very deserted.” (participant 7, male, age 31–40)*

##### Training and workshops

There was also a need on the part of the participants for training courses and workshops, both online and on-site, on a wide range of topics. E.g., it was mentioned that as hybrid work is a new form of collaboration, training courses are needed to teach employees how to work hybrid. This would primarily involve the conveyance of technical knowledge, but also social and personal skills in handling the hybrid situation as simply experiencing hybrid work may not be sufficient in the learning process. Other topics in this regard could include behavior in online meetings or the independent organization and management of work:

*“[…] that was what I meant earlier, with training, further training, maybe also for myself […], I would also wish for: How do I handle 20 digital meetings a week? Or how do I organize myself? I think we all have to learn that now.” (participant 5, male, age 41–50)*

In the course of this, care should specifically be taken to support less technologically literate people in particular, so that they can adjust to hybrid work. Other desires related to more frequent workshops that cater to both professional and teambuilding aspects. Topics could, e.g., include resilience or work-life balance. Another possibility that was referred to concerns best-practice presentations. Here, departments of the authority in which hybrid work and collaboration are successful could introduce themselves to other departments and report on their experiences.

##### Strategies and self-discipline for boundaries and structure

The interviewees also mentioned the need to acquire strategies and the necessary motivation to create stronger boundaries in hybrid work between private life and work. Reported strategies were to schedule fixed appointments in the own calendar for break times or to avoid eating lunch at the workplace while continuing to work:

*“For myself, I should just pay more attention to the real separation between office work and free time. So eating lunch at the workplace and then still doing something on the side is simply a question of self-discipline.” (participant 4, female, age 51–60)*

According to participants, separate office rooms or fixed working areas as well as set routines (e.g., putting on or taking off shoes at the beginning or end of work) would also help to set boundaries in the home office. As a team, meetings could generally be scheduled with short breaks in between. Proposed strategies for a better structure included set work times, e.g., starting and finishing work at the same time every day, stowing away the work equipment at the end of the day, and prioritizing work tasks. A general awareness should be created to refrain from being available at all times and to the fact that boundaries are blurring:

*“[...] I think that setting boundaries for yourself is a very important point, [...] also in the form of: 'I'm not going to answer the phone right now'. But it's a very difficult thing, so I think you really have to do a lot of convincing or be very strong for yourself to say, 'I'm definitely not going to answer it now because I have to finish this and that.'.“ (participant 1, female, age 21–30)*

## Discussion

This study applied qualitative methods to provide important insight into first-hand perceptions and experiences of job demands, job resources, and support needs of employees who work hybrid in German public administration. Qualitative research grounded on the JD-R model has the potential to identify relevant factors that possibly function as job demands and resources in rather unexplored fields of work in the first place (cf. [31]).

### Job demands, resources, and support needs in hybrid work

With regard to our first research question, this is to our knowledge one of the first studies to have comprehensively captured the job demands and resources that arise from the interplay of the remote and office work components in the hybrid context in public administration. A study within Swedish public administration [37] elaborated on anticipated challenges and opportunities in the hybrid work environment while our study considers the actual experiences in hybrid work. Referring to the second research question of this paper, we identified a multitude of support needs on the structural and behavioral level. The structural level support needs emphasize the need to hold employer and management levels in public administration accountable for establishing healthier working conditions in hybrid work. While some of our findings are consistent with previous literature on hybrid work, we were also able to identify novel nuances of perceived job demands, resources and support needs.

We identified a range of job demands and resources that referred to all of the six GDA domains (1. *work contents and tasks*, 2. *work organization*, 3. *work time*, 4. *social relationships*, 5. *work equipment*, and 6. *work environment*). Although the GDA domains initially seemed to comprehensively capture the types of work characteristics to be considered when assessing and preventing mental stress in work contexts, our study identified an additional characteristic – *organizational resources.* By providing workplace health management and promotion offerings for employees (e.g., digital exercise programs), the public administration organizations offered interviewees an additional opportunity to support them in their work-related and personal development.

A multitude of the participants' further experiences related to *work organization* and *social relationships*. Particularly noteworthy in terms of *work organization* is the observation that management levels in public administration still seem to reject hybrid work to a certain degree, although, during the period where the interviews were conducted, hybrid working arrangements had been implemented for some time. The participants primarily experienced this by managers' lack of endorsement of the home office component. This discrepancy between employers' and employees' preferences towards hybrid work was earlier brought to light by a study conducted by Aksoy et al. [23]. Surveyed German employees on average reported that their employers allowed them to work from home on 0.7 days per week whereas the employees themselves would on average prefer a higher number of 1.6 home office days [23]. A job resource and decisive factor for the success of hybrid work for the participants was thus found in the ability of the management and employees to adapt to varying work procedures and needs in hybrid work. In line with this, participants emphasized the need for more acceptance and promotion of hybrid work on the part of the management and the development of mutual understanding and accommodation between managers and employees. A comprehensive acceptance of hybrid work throughout the organization essentially represents a gatekeeper for effectively addressing the support needs in hybrid work. The sufficient advocation of stakeholders in question seems particularly necessary to initiate the implementation of appropriate measures that respond to the various support needs of employees.

Anticipated from the outset, another highly pertinent job demand which we identified for hybrid work is the limited communication in digital and hybrid meetings. The occurrence of the observed challenges appeared predictable due to the virtuality aspects that come with digital and hybrid communication which can impact trust building in teams due to disruptions or misunderstandings [56]. Be it in video conferences or via e-mail, less exchange of emotions reactions and body language takes place in virtual compared to face-to-face communication [56]. Although teams that work in a hybrid mode are not fully dependent on virtual communication, they are still confronted with comparable challenges*.* Interestingly, in this study we nonetheless identified that virtual communication in hybrid work also comes with benefits such as increased efficiency, which has the potential to complement the advantages of face-to-face communication, altogether fostering a job resource of successful communication and collaboration in hybrid work. Concerning face-to-face communication, part of the participants either experienced joint office attendance times for team members as a job resource or as a support need. Additionally, the interviewees rated the maintenance of personal face-to-face contact and interpersonal relationships with co-workers – online and in person – as a crucial resource which also aligns with previous findings on enhanced belonging, socializing, spontaneity and creativity when working and communicating hybrid [37, 41] instead of purely virtual. This is precisely why a balanced blend of both work locations in hybrid work should ideally offer a significant advantage for collaboration. However, as the persistence of job demands in, e.g., a limited talking culture illustrates, more department or team-specific agreements might be necessary to create this optimal balance in some cases.

Although hybrid work has, at the time of this study, been implemented for a while, our study confirmed the existence of hurdles for the transformation to hybrid work in public administration that we expected based on current evidence. In terms of *social relationships*, e.g., the culture of presence [8], appears to persist, as interviewees in our study described the pressure to attend the office on-site that is exerted in hybrid work as a job demand. This only validates previous study results in which around half of public administration employees, for instance, agreed that not being on-site can be a hindrance to the career or that being on-site is important to show that work is being done [8]. It therefore seems reasonable that the employees that participated in our interviews proposed a specific support need for a change in management style towards management by objectives or other results-oriented work approaches. Results-oriented management styles would measure employee productivity based on their actual achievement of work results [57] rather than based on the amount of time that they spend on-site.

As another anticipated challenge for public administration organizations' transformation to hybrid, further job demands arose regarding the technical infrastructure and equipment which some of the interviewees reported to be missing, inadequate or misused. This has previously been highlighted in other remote public administration contexts [7, 37] where the technical infrastructure did not suffice for remote work opportunities and collaboration tools or mobile devices in particular were lacking [7]. Specific to the hybrid work context we were, however, able to identify a support need relating to technical equipment that allows for inclusivity in hybrid meetings that combine co-located and virtually joined participants. In a study on communication practices in hybrid meetings, Saatçi et al. [58] propose that the currently used video communication technologies fail to meet the needs of hybrid meetings, not allowing cross-location engagement. Said realization underlines the need to design and adapt the technical equipment to meet the individual needs of different meetings. This could for instance be realized by pre-setting meeting configurations that, e.g., technically define an order of talk which is then made visible for each of the meeting participants in advance and during [58].

### Context dependency and subjectivity of job demands and resources in hybrid work

An overarching view of the identified job demands and resources raises some striking contradictions. The flexibility of the JD-R model can compromise the specificity of the derived demands and resources which Bakker and Demerouti [28] consider to be the model's "Achilles' heel": certain job characteristics can act as either a resource or a demand depending on the work context. So, while in some positions, interviewees, e.g., saw a demand in the lack of social contact and exchange within hybrid work, others rather deemed the distance to managers and co-workers as healthy and a job resource. Discrepancies could also possibly be the result of subjectivity in the perception of working conditions and the individuality of work behaviors: while some participants reported on their successful preservation of boundaries and structure in hybrid work, others experienced a lack of said aspects. They wished to either acquire skills and the necessary self-discipline to create stronger boundaries and structure in their work lives or for the management level to create the corresponding awareness. Such discrepancies may be attributable to the existence of different work-life boundary management styles as in ways that people approach demarcating their work and private lives [59].

At the same time, discrepancies may also arise because some of the voiced resources or demands relate solely to the remote or on-site component of hybrid work and the respective opposite component is used for reference when participants classify a work characteristic as rather positive or negative. Opposed to the interviewees who experienced a lack of social contact and exchange due to co-workers being dislocated, others also emphasized that personal contact was maintained due to the in-presence component of hybrid work. So, while hybrid work finds itself on a fluid spectrum between remote and office work, the respective component that the interviewee has in mind at the time of his judgement could influence whether they perceive a specific work characteristic rather as a job demand or resource of hybrid work. Job resources of the on-site component of hybrid work could in a sense be understood as the counterparts of the demands in remote work and vice versa.

Incongruencies regarding hybrid work conditions are also apparent in the current evidence. Regarding, e.g., productivity and efficiency, studies on the one hand revealed an increase due to fewer interruptions or an improved work-life balance [18, 39, 41]. Other studies, however, report deficiencies in productivity, motivation, and engagement in hybrid work [37, 39, 40].

In terms of context dependency, it also plays a role that the study is set in Germany, where hybrid working conditions and the associated demands and resources may differ from those in other countries. Our observations generally underline the diversity of individual employees, workplaces, and departments in public administration and show that the current degree of implementation regarding health-promoting working conditions strongly varies. When deriving implications, it should therefore be refrained from solely standardizing the results to the entire public administration. Instead, value should be placed on needs-based solutions for individual departments and employees.

### Research implications

While the explorative approach provided an initial overview of job demands and resources, their actual prevalence among hybrid workers in public administration and their impact on potential employee outcomes should be further investigated in the future. Further quantitative studies should focus on specifically analyzing health-related outcomes such as burnout, absence duration, or well-being (cf. [28]). Corresponding to occupational stress models such as the JD-R model, data analyses could allow investigating correlations, interactions of different job demands and resources, and their effects on specific outcomes. Quantitative research could further enable comparisons of existing job demands and resources between different employee groups. For instance, an examination of how certain aspects affect women and men or different occupational groups differently, or how differences might arise depending on individual employees' balance of remote and on-site work.

While this study concentrated on the hybrid work experiences of employees, another relevant perspective would be the one of stakeholders at management level in public administration. Initial exploratory studies could serve to discover the difficulties or opportunities that they perceive in hybrid work. Subsequently, it could also be investigated in how far supervisors' job demands and resources resemble or contradict those of employees. Deriving corresponding measures for managers could result in them role modeling health-promoting behaviors in hybrid work which would then again have positive effects on the employees' health.

### Practical implications

The results of this study build a basis for a needs-based design of health-promoting working conditions [27] in the context of hybrid work in general and the field of public administration in particular. The overarching aim for practice should be to 1. generally minimize the reported negative levels of job demands and to expand the job resources, and 2. respond to the support needs and opportunities on the behavioral and structural level identified within this study.

The realization of health-promoting structures in hybrid work contexts requires the participation of different organizational stakeholders. To respond to structural needs, the employer and different levels of management need to be involved and should implement measures that improve organizational and leadership culture and the working conditions of employees in hybrid work (cf. [35, 36]). As one of the key strategies, the employer and managers should strengthen their acceptance of the hybrid working model and establish a positive image, as this serves as a determining facilitator for further measures that ensure health-promoting hybrid working conditions. Along with this, importance goes to the adoption of leadership strategies adapted to hybrid work such as establishing flexibility at the workplace, and putting trust into the employees' decisions [60, 61]. Corresponding responsibilities should be incorporated in organizational regulations and guidelines that ensure ways of how hybrid work can be accepted and promoted, or how greater boundaries can be encouraged. The results also showed that sources of support in hybrid work can vary and already exist in some parts of public administration, e.g., psychosocial counseling services. An expansion of the already existing structures is therefore of equal relevance.

Behavioral level support needs mostly necessitate a change or adoption of work behaviors and habits on the individual employee's side (cf. [35, 36]), e.g., a reinforcement of strategies and self-discipline for boundaries and structure in hybrid work. Employees who still experience difficulties in boundary and structure management should learn and be taught strategies to demarcate their work and private life. Strategies could consist of planning transition times between work and private life activities (e.g., by refraining from doing work tasks on the commute) or organizing specific time blocks for focused work [59]. While behavioral level needs specifically require the employees to be involved, corresponding measures should not solely be left to the employees to implement. Instead, the employer and direct managers are also called upon as stakeholders. To support the different boundary needs of employees, managers, and employers can make use of strategies such as emotional support or setting examples as a role model [59] or by providing corresponding learning offers such as workshops.

The practical implications may not be generalizable in an international context as cultural differences have the potential to produce completely different results. However, even at the regional level of this study, the results showed that employees' perceptions of working conditions and support needs can vary. Certain measures may already be implemented in some departments and may be more necessary in other departments of public administration. This highlights the importance of conducting department-specific enquiries, e.g., via employee surveys. Joint bilateral or small group consultations between employees and their supervisors to develop mutual understanding could also be helpful in order to identify individual needs in hybrid work. Altogether, a succinct challenge in hybrid work probably forms finding a balance between remote and office work that allows employees to profit from a broad variety of advantages that each of the work environments offers, while also mitigating the drawbacks of each to the greatest possible extent. For an optimal balance of remote and office work and a sustainable implementation of health-promoting hybrid working conditions in general, it is, however, essential to not only consider individual employee needs but harmonize the needs on the individual, departmental, and authority levels (cf. [62]). This emphasizes the relevance of exploring the experiences in hybrid work of organizational leaders as stated in the research implications.

### Strengths and limitations

This study is accompanied by strengths, such as that job demands and resources within hybrid work were recorded comprehensively while putting a special focus on public administration in Germany. The JD-R model provided an appropriate and empirically based theoretical framework to guide the exploratory method of this study. Utilizing the GDA domains, we were able to systematically categorize and overarchingly compare the identified resources and demands. Within the sample selection, an even distribution regarding gender was achieved. By following the principle of data saturation, it was ensured that relevant experiences and perceptions of affected employees were included to their full extent.

Although reaching data saturation, the small sample size (*n* = 13) should be discussed among the limitations of this study as it is accompanied by a restricted representativeness of different employee characteristics. The majority (*n* = 10) have only been working in their position for three years or less. Consequently, the perspectives of long-standing employees who have been accustomed to a non-hybrid work environment in their current position for many years and for whom the switch to hybrid work represents a substantial change was underrepresented. In addition, only one participant reported to visit the office on more than half their work time. The sample was therefore skewed to the extent that the majority of respondents worked remotely very frequently. It is conceivable that individuals in favor of remote work may tend to report more job resources and fewer job demands in hybrid work, in contrast to those holding a more negative attitude toward remote work. Lastly, the sample had a high representation of employees in the digital or information technology sector, which again may come along with higher advocacy of hybrid work as it is usually accompanied by an increase in digital work.

With the COVID-19 pandemic as a catalyst for the spread of hybrid working, it is also important to consider the temporal context within which the interviewees' conveyed their reported experiences. Even though the interviews themselves were conducted around the time when the protection measures officially expired in Germany, the interviewees' narratives were notably influenced by their prior experiences during the abrupt transition to remote work at the onset of the pandemic. For example, participants emphasized certain factors, such as the limited talking culture, as being particularly challenging at the start of the pandemic.

When looking at the results, not all factors relate directly to the interaction between the office and remote work components that is characteristic to hybrid work, but in some cases only to one of the components. The resource of saving travel distances to the office, e.g., only refers to working from home. However, as both the office and remote work individually are inherent parts of hybrid work, job demands and resources at the individual component level also need to be taken into account. Partial results could therefore also be applied to the singular contexts of remote or office work.

## Conclusions

This study contributes to a comprehensive exploration of the existing job demands and resources in hybrid work and prepares grounds for measures in hybrid work contexts that prevent mental stress and promote the health of employees. A large number of perceived job resources as well as job demands in the hybrid context were reported in the domains of *work organization* and *social relationships*. In some cases, we found that job demands and resources contradicted each other which for instance may be a result of subjectivity in employees' different preferences in hybrid work. The results of this study make an essential contribution to further necessary research into hybrid working conditions and their effects on (different groups of) employees. Based on their experienced job demands and resources, the participants expressed a variety of needs for support in hybrid work at both the structural and behavioral level. This indicates that different levels of organizational stakeholders, including the public employer, management and individual employees, need to be involved in the implementation of corresponding measures. Similar to the experiences of job demands and resources in the hybrid context, support needs can be subjective. In practice, it is relevant to search for solutions that balance the individual needs of employees, and the interests of the workplace on departmental and authority level. To create the organizational motivation to implement corresponding strategies, acceptance and promotion of hybrid work throughout public administration bodies is necessary.

Hybrid work is still in its early stages but will presumably not disappear. Public administration employers should therefore act as early as possible to ensure coverage of the various needs and to realize health-promoting working conditions in the ongoing transition to hybrid work. The findings, and along with them the practical implications that were derived in this study, provide an important first point of reference.

### Supplementary Information

Supplementary Material 1.

## Data Availability

The datasets generated and/or analyzed during the current study are not publicly available due to German national data protection regulations but are available from the corresponding author on reasonable request.

## Abbreviations

COREQ: Consolidated criteria for reporting qualitative research
GDA: Joint German Occupational Safety and Health Strategy
JD-R: Job demands-resources
PCI: Problem-centered interview
