# Supplementary material for: Job demands and resources perceived by hybrid working employees in German public administration: a qualitative study
Source: J Occup Med Toxicol. 2024 Jul 19;19:28. doi: 10.1186/s12995-024-00426-5 (PMC11264583; doi:10.1186/s12995-024-00426-5)
Supplement: Supplementary file 1 — Supplementary Material 1. [file 12995_2024_426_MOESM1_ESM.pdf]

Additional file 1: Structure of the interview guide used in problem-centered interviews

Interview phase	Communication strategies	Contents
Introductory phase	-	▪ Information on the study<br>▪ Theoretical introduction to the topic of hybrid work<br>▪ Standardized short questionnaire on sociodemographics, work experience, job-related information
1. Job demands and resources in hybrid work	Strategies to generate storytelling	*Introductory question:*<br>▪ “Is the possibility of hybrid working a curse or a blessing for you, or perhaps both? Why?”<br>*General exploration, open questions:*<br>▪ Open questions about demands and resources (e.g., aspects of hybrid work that have a demotivating effect and aspects that bring pleasure)<br>*Ad-hoc questions:*<br>▪ Questions on specific areas e.g., communication and cooperation, social support, technology or flexibility and control
	Strategies to generate comprehension	▪ Reflection of the participants' previous statements
2. Support needs and opportunities for improvement in hybrid work	Strategies to generate storytelling	*General exploration, open questions:*<br>▪ Open questions about aspects which could help in hybrid work<br>▪ Ways of expanding the resources or reducing the demands<br>*Ad-hoc questions:*<br>▪ Questions about support needs at the individual, team or management level
	Strategies to generate comprehension	▪ Reflection of the participants' previous statements<br>▪ Inquiries about support and improvement possibilities to prevent or expand the demands and resources that the participants discussed in interview phase 1
